# Developmental Acquisition of a Rapid Calcium-Regulated Vesicle Supply Allows Sustained High Rates of Exocytosis in Auditory Hair Cells

**DOI:** 10.1371/journal.pone.0025714

**Published:** 2011-10-06

**Authors:** Snezana Levic, Yohan Bouleau, Didier Dulon

**Affiliations:** Equipe Neurophysiologie de la Synapse Auditive, Unité Mixte de Recherche, Inserm U587 et Université Victor Segalen, Institut des Neurosciences de Bordeaux, Centre Hospitalier Universitaire Pellegrin, Bordeaux, France; Dalhousie University, Canada

## Abstract

Auditory hair cells (HCs) have the remarkable property to indefinitely sustain high rates of synaptic vesicle release during ongoing sound stimulation. The mechanisms of vesicle supply that allow such indefatigable exocytosis at the ribbon active zone remain largely unknown. To address this issue, we characterized the kinetics of vesicle recruitment and release in developing chick auditory HCs. Experiments were done using the intact chick basilar papilla from E10 (embryonic day 10) to P2 (two days post-hatch) by monitoring changes in membrane capacitance and Ca^2+^ currents during various voltage stimulations. Compared to immature pre-hearing HCs (E10-E12), mature post-hearing HCs (E18-P2) can steadily mobilize a larger readily releasable pool (RRP) of vesicles with faster kinetics and higher Ca^2+^ efficiency. As assessed by varying the inter-pulse interval of a 100 ms paired-pulse depolarization protocol, the kinetics of RRP replenishment were found much faster in mature HCs. Unlike mature HCs, exocytosis in immature HCs showed large depression during repetitive stimulations. Remarkably, when the intracellular concentration of EGTA was raised from 0.5 to 2 mM, the paired-pulse depression level remained unchanged in immature HCs but was drastically increased in mature HCs, indicating that the Ca^2+^ sensitivity of the vesicle replenishment process increases during maturation. Concomitantly, the immunoreactivity of the calcium sensor otoferlin and the number of ribbons at the HC plasma membrane largely increased, reaching a maximum level at E18-P2. Our results suggest that the efficient Ca^2+^-dependent vesicle release and supply in mature HCs essentially rely on the concomitant engagement of synaptic ribbons and otoferlin at the plasma membrane.

## Introduction

The ribbon synapse of cochlear hair cells (HCs) encodes sound information by tightly controlling the number (discharge rate) and precise timing (temporal coding) of postsynaptic spikes. Remarkably, this synapse can drive postsynaptic auditory nerve fibers at extremely high instantaneous discharge rates (over several thousand spikes/s at stimulus onset) and, after rapid adaptation, can support sustained discharge rates over several hundred spikes/s during ongoing sound stimulation [1], [2]. To sustain such high rates of synaptic exocytosis, auditory HCs must have efficient mechanisms to rapidly and constantly replenish the pool of synaptic vesicles. While it is well established that the rate of vesicle fusion is tightly controlled by Ca^2+^ ions flowing through nearby voltage-gated Ca^2+^ channels [3], little is known about the mechanisms regulating the kinetics of vesicle supply at the ribbon active zone. Recently, it was hypothesized that otoferlin, a multi-C2 Ca^2+^ sensor that directly regulates SNARE-membrane fusion *in vitro* [4] and is required for HC vesicle exocytosis [5], also controls the supply of synaptic vesicles at the active zones [6]. Surprisingly, during their early developmental period, immature auditory HCs transiently express several Ca^2+^-dependent synaptotagmins and do not require otoferlin to control phasic transmitter release driven by spontaneous action potentials [7]. With cochlear maturation, the Ca^2+^ efficiency and kinetics of exocytosis in HCs largely increase [8], [9], [10], as well as the expression level of otoferlin in HCs [5], [7]. However, the precise mechanisms of how otoferlin is engaged to produce fast vesicle supply and release in developing HCs remains to be elucidated.

Notably, the first sound-evoked responses of immature auditory nerve fibers in the developing cochlea display high thresholds of rhythmic bursting activity and are unable to maintain a sustained steady-state response to long duration tone bursts [11]. The present study tests the hypothesis that neurotransmitter availability is an important factor that limits sustained postsynaptic firing activity in developing auditory afferent neurons. By studying when the ability to produce sustained high rate of exocytosis is acquired by HCs during development, we attempt to gain insights into the mechanisms of synaptic vesicle replenishment. In chick embryo, the first indication of sound-evoked electrical responses from the inner ear have been reported from the 11th day of incubation (E11) [12], [13], [14]. The auditory thresholds then show continuous maturation between E15 and the first post-hatching day (P1) to attain adult values. In the present study, we took advantage of the slow maturation of the auditory chick basilar papilla to characterize the progressive changes occurring in exocytosis and vesicle supply at the HC ribbon synapse.

## Materials and Methods

### Preparation of semi-intact chicken basilar papilla

The present investigation was performed in accordance with the guidelines of the Animal Care and Use Committee of the European Communities Council Directive of November 24^th^, 1986 (86/609/EEC) and the University of Bordeaux (ethics committee: Direction Régionale de l'Alimentation, de l'Agriculture et de la Forêt d'Aquitaine (DRAAF Aquitaine) permit number B 33075, approved this study). The study included chickens at different stages of embryonic development ranging from E10-E21 as well as two-day post-hatched chickens. Fertilized eggs were incubated at 37°C in a Marsh automatic incubator (Lyon Electric, Chula Vista, CA). Chicken embryos were sacrificed and staged according to their number of somites, and additionally for the late stages as following: from E8-E12 based on visceral arches, feather gems and eyelids: after E12 based on the length of the beak [15]. Basilar papillae were isolated as described previously [16]. The preparations were dissected in oxygenated chicken saline containing (in mM) 155 NaCl, 6 KCl, 4 CaCl_2_, 2 MgCl_2_, 5 Hepes, and 3 glucose, pH 7.4. The tegmentum vasculosum and the tectorial membrane were removed without any prior enzymatic treatment using a fine minutia needle. Chicken basilar papillae were stored in a 37°C incubator in Minimum Essential Medium (Invitrogen) before recordings from HC *in situ*. All experiments were performed at room temperature (21–23°C) within 5–45 min of isolation. All reagents were obtained from Sigma Chemicals, unless otherwise specified. Recordings were done in neural (tall) HCs along the basilar papilla. These HCs which are mainly innervated by the afferent fibers correspond to the inner hair cells in mammals. The tonotopic location of HCs was divided in three parts. From the proximal narrow end of the papilla, the first 1/3 part was considered as the high frequency coding region of basal HCs. Low frequency apical HCs were recorded at the top 1/3 part of the wide end of the basilar papilla.

### Electrophysiology

Calcium currents were recorded in whole-cell voltage-clamp configuration using 3–5 MΩ resistance pipettes. Currents were recorded with an EPC 10 amplifier (Heka Electronik, Lambrecht/Pfalz, Germany) and filtered at a frequency of 2–5 kHz through a low-pass Bessel filter. The sampling frequency was determined by the protocol used. No online leak current subtraction was made. Only recordings with holding current less than 20 pA were accepted for analyses.

Real-time changes in membrane capacitance (ΔC_m_) were recorded using the EPC 10 amplifier. A 2 kHz sine wave of 10 mV was applied to the cells from a holding potential of -90 mV. Capacitance (Cm) signals were low-pass filtered at 80 Hz. Changes in membrane capacitance were measured 0.05–0.5 s after the end of the depolarizing pulse and averaged over a period of 0.2–2 s. Membrane and series resistance (R_m_ and R_s_) were monitored during the course of the experiment. Only recordings with stable R_m_ and R_s_ were considered for further analysis. The study included ∼247 cells with R_s_ within the 5–20 MΩ range. Holding membrane potentials were corrected for liquid junction potentials. Extracellular solution for measuring Ca^2+^ currents contained (in mM) NaCl/CholineCl 125, KCl 6, CaCl_2_ 5, 25 TEA, 5 4-AP, D-glucose 10, MgCl_2_ 1, HEPES 10, pH 7.3, 310 mOsm. TEA and 4-AP were present in the external medium to block residual inward rectifying K^+^-currents that may contaminate the measured inward Ca^2+^ current. Intracellular solution contained (in mM) NMG 75, CsCl 70, Na_2_ATP 5, MgCl_2_ 2, HEPES 10, EGTA 0.5–10, and glucose 10; pH 7.3, 300 mOsm.

### Immunohistochemistry

Tissues were fixed with 4% paraformaldehyde in PBS (PBS) for ∼3 hrs, then rinsed and immunostained with a polyclonal antibody directed against mouse CtBP2 (1∶200, Sigma) and a monoclonal HCS1-antibody (1∶250, a gift from Dr Jeffrey Corwin, University of Virginia, [17]. Immunostaining was visualized with anti-goat secondary antibody conjugated to Alexa 488 (green, CtBP2) and anti-mouse secondary antibody conjugated Alexa 546 (Red, HCS1). Omission of the primary antibodies eliminated staining in all preparations examined. HC actin was counterstained with phalloidin conjugated to Alexa Fluor 647 (1∶200, Molecular Probes (Invitrogen, Carlsbud, CA). Fluorescent images were collected and analyzed with a confocal laser scanning upright microscope (Leica DMR TCS SP2 AOBS, Bordeaux Imaging Center). Images of ribbons were taken in the basal synaptic area of the HCs (step size 0.4 µm).

### Data analysis

The number of cells (n) is given with each data set. Data were analyzed using pClamp10 (Axon Instruments) and Origin7.0 (Microcal Software). Pooled data were presented as mean ± SD. Significant difference between groups of cells or between different embryonic stages of development was evaluated using a two-tailed Student's t test; *p* values are presented in the text and figure to indicate statistical significance. Time constants (τ_s_) were obtained from fits using Origin software. Time constants were obtained by fitting multiple exponential equations to the activation decay of the current. The equation was of the form:
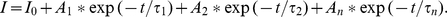
Where I_0_ is the initial current magnitude, τ_1_, τ_2_...τ_n_ are the time constants, and A_1_, A_2_...A_n_, are the proportionality constants. Synaptic transfer functions relating Ca^2+^ current (I_Ca_) and ΔCm, or Q_Ca_2+ and ΔC_m_ were calculated using an integral of total I_Ca_, including the tail currents. The data was fitted using first-order power functions: 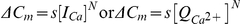
where s  =  slope factor (fF/pA or fF/pC), and N  =  power index.

The % RRP refilling was calculated as:
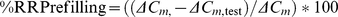
where ΔC_m_ = ΔC_m_ measured using the first, control pulse, and ΔC_m, test_ = ΔC_m_ measured using test pulse.

The % of I_recovered_ was calculated as:
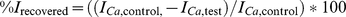
Where I_Ca, control,_ = I_Ca_ measured using the first, control pulse, and I_Ca, test_ = I_Ca, test_ measured using test pulse.

## Results

### Kinetics and Ca^2+^-efficiency of RRP exocytosis increase with cochlear maturation

The efficiency of Ca^2+^-evoked exocytosis was characterized at four developmental periods of cochlear synaptogenesis: embryonic stages (*in ovo*) E10–11, E12–14, E16–18 and 2 days post-hatching P2. The first embryonic period, E10–11, corresponds to an early stage of synaptogenesis when the first presynaptic specializations (synaptic bodies or ribbons) can be detected in HCs [18] and when the afferent fibers first contact their base [19]. At stage E11–E14 low frequency hearing starts in the chick embryo [12], [13], [14]. Stage E16–E18 (5-3 days before hatching) corresponds to the final step of synaptogenesis and HC maturation. Finally, P2 corresponds to nearly adult hearing values [20].

**Figure 1:**
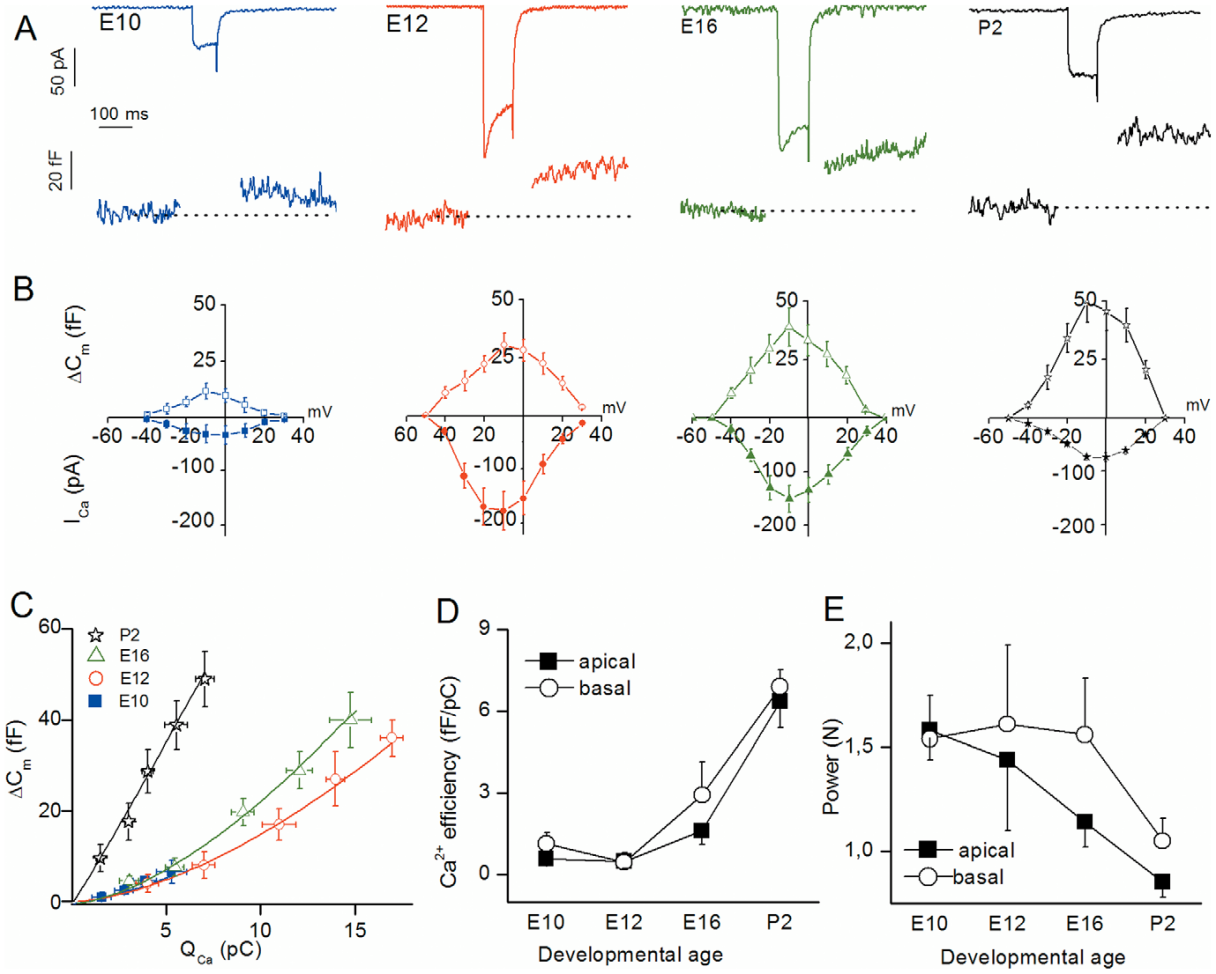
Ca^2+^ efficiency of RRP exocytosis increases with maturation. (A) Examples of I_Ca_ and ΔC_m_ recordings following a 100 ms-voltage step from holding potential of -90 mV to -10 mV at E10, E12, E16, and P2 basal HCs. (B) Voltage-dependence of I_Ca_ and ΔC_m_ recorded in basal HCs at E10 (n = 6), E12 (n = 9), E16 (n = 9) and P2 (n = 7). I_Ca_ was measured at its peak value during the voltage pulse shown in A. To limit depression, each consecutive voltage-step was separated by a 30 s recovery period. (C) Synaptic transfer functions relating Q_Ca_ (charge integral of Ca^2+^ current) and ΔC_m_ in basal HCs. Data points were fitted using first order power function with ΔC_m_ = s[I_Ca_]*^N^*, where s =  slope factor (Ca^2+^ efficiency; fF/pC) and *N* =  power index or degree of co-operativity. Values of s and N are reported in table 1. (D) Comparative Ca^2+^ efficiency (ΔC_m_/Q_Ca_) in apical (E10, n = 10; E12, n = 13; E16, n = 11; and P2, n = 9) and basal HCs (n as in B) as a function of developmental age. (E) Comparative cooperative (power) index *N* from power fit of data in D as a function of age in developing apical and basal HCs.

At all developmental stages from E10 to P2, rapidly activating inward current (I_Ca_) and a concomitant increase in membrane capacitance (ΔC_m_) was recorded when HCs were voltage-stepped from -90 mV to varying depolarized potentials (Fig. 1A–1B). The voltage-activation curve of ΔC_m_ displayed a bell shape that followed the I_Ca_ activation curve (maximum amplitude near -10 mV), a behavior consistent with ΔC_m_ being activated consecutive to Ca^2+^ influx. Indeed, complete blockage of I_Ca_ by 250 µM Cd^2+^ eliminated ΔC_m_ (data not shown), confirming that ΔC_m_ is sensitive to Ca^2+^ entry via VGCC, in agreement with [21]. For a 100 ms-depolarization, which is considered to entirely release the readily releasable pool (RRP) [22], the amplitude of ΔC_m_ responses increased with HC maturation (Fig 1A–B). In low frequency apical HCs, ΔC_m_ responses were as follows: (in fF at -10 mV) E10, 10±2 (n = 10); E12, 28±6 (n = 13); E16, 37±7 (n = 11); and P2, 45±6 (n = 9). High frequency HCs recorded at the base of the basilar papilla were also found to undergo a similar increase in ΔC_m_ responses with maturation (Fig. 1A–B and table 1). Notably, at embryonic stages earlier than E10, while activating significant I_Ca_ (17.5±3.1 pA at -10 mV, n = 7, E7–E8), 100-ms voltage-step depolarization did not produce significant ΔC_m_ responses in all HCs tested (below background level of 3.8±1.3 fF; data not shown).

**Table 1:** Main characteristics of Ca^2+^ dependence of exocytosis in developing chick HCs (LF =  low frequency and HF =  high frequency).

Exocytosis	E10	E12	E16	P2
***Ca^2+^ dependence (N)***				
Apical (LF)	**1.58±0.14**	**1.44±0.34**	**1.14±0.12**	**0.85±0.07**
Basal (HF)	**1.54±0.21**	**1.61±0.38**	**1.56±0.27**	**1.05±0.11**
***Ca^2+^ efficiency (fF/pC)***				
Apical (LF)	**0.58±0.13**	**0.49±0.27**	**1.6±0.5**	**6.35±0.97**
Basal (HF)	**1.14±0.4**	**0.45±0.35**	**2.92±1.2**	**6.89±0.63**
***RRP release rate (vesicle/s)***				
Apical (LF)	**3158±698**	**10198±2245**	**18318±3253**	**30540±4122**
Basal (HF)	**3720±1126**	**11393±1828**	**22015±4623**	**38687±4460**
***Total vesicles in RRP (100 ms)***				
Apical (LF)	**281±62**	**764±168**	**989±176**	**1221±164**
Basal (HF)	**316±96**	**827±133**	**1056±221**	**1354±153**

N indicates power (cooperative) index.

The synaptic transfer function relating ΔC_m_ as a function of charge entry (Q_Ca_ as time integral of I_Ca_) was compared at different developmental stages by stepping the cells to various potentials from -60 to -10 mV for a constant 100 ms duration (Fig.1C). With maturation, data points both in apical and basal HCs were fitted by first-order power functions with decreasing power index (N; cooperative index) and increasing slope factors (Ca^2+^ efficiency, fF/pC) (Fig. 1D–E; table 1). These results indicated that, similarly to mouse cochlear HCs [8], [9], maturation of the chick HC synapse is associated with a better coupling between Ca^2+^ influx and vesicular release.

**Figure 2:**
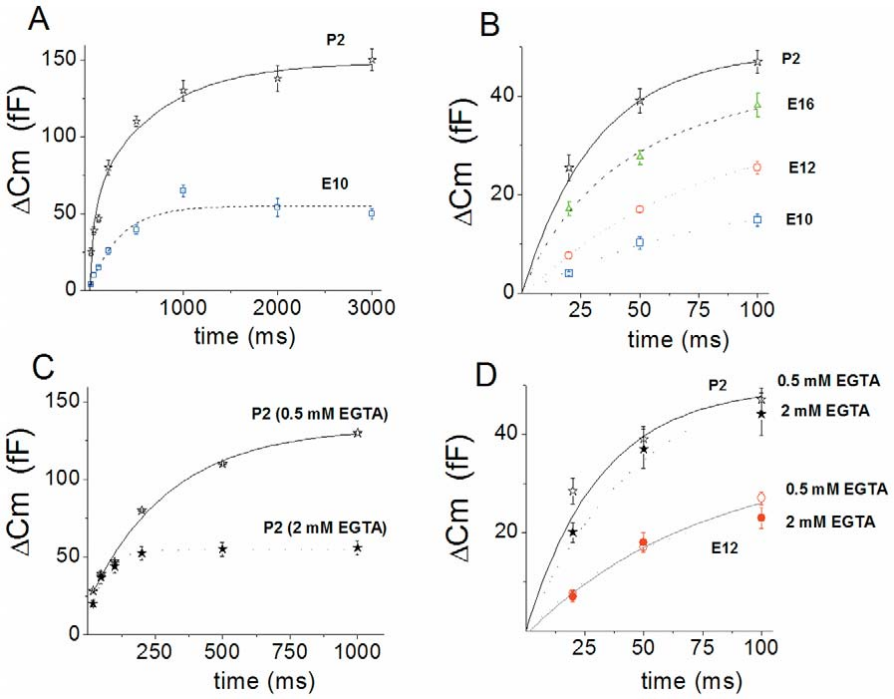
Kinetics of vesicle release increases with HC maturation. Cochlear apical HCs were voltage-stepped from holding potential of −90 mV to -10 mV for increasing duration (20 to 3000 ms). To avoid synaptic depression, each pulse of varying duration was separated by a 30 s recovery period. A) Comparative mean ΔC_m_ responses (RRP+SRP) recorded in P2 (n = 8) and E10 (n = 6) apical HCs. B) RRP depletion, corresponding to the first 100 ms of release, was fitted with a single exponential with τ = 89, 75, 48, and 39 ms at E10 (n = 6), E12 (n = 7), E16 (n = 9) and P2 (n = 8), respectively. (C–D) Comparative ΔC_m_ responses (RRP + SRP) recorded in P2 or E12 apical HCs using 0.5 (as in A and B) or 2 mM intracellular EGTA (n = 4 and 5, respectively). Contrary to the SRP, note that the RRP (D) is not affected by 2 mM EGTA in both mature and immature HCs.

Changes in release rate were then compared by stepping HCs to constant potential (from -90 to -10 mV) for different durations from 20 to 3000 ms (Fig. 2). Data points were best fitted by two exponential functions that likely described a fast release of a readily releasable pool (RRP) of vesicles (up to 100 ms) and a secondary, slowly releasable pool (SRP) as previously described [9], [21], [23]. Kinetics of RRP release largely increased with maturation from E10 to P2, with respective time constants of RRP release corresponding to 89±7 ms (n = 10) and 39±4 ms (n = 9), (*p<0.001*, Fig.2B). Accordingly, the RRP release rate (vesicles/s) increased from 3,158±698 at E10 to 30,540±4122 at P2 in apical HCs (*p<0.001*; table 1). The SRP amplitude also increased nearly 3-fold from E10 to P2 (Fig. 2B). By varying the concentration of the Ca^2+^ buffer EGTA from 0.5 (estimated buffer space of ∼ 1000 nm) to 2 mM (estimated buffer space of ∼ 200 nm from the point of Ca^2+^ source) [22], the kinetics of RRP release were unaffected both in immature E12 HCs and in mature P2 HCs (Fig. 2D). However, as expected for the recruitment of vesicle located farther than 200 nm from Ca^2+^ entry, the SRP was largely reduced by 2 mM EGTA (Fig. 2C).

### The rate and Ca^2+^ sensitivity of vesicle supply to the RRP increase during development

**Figure 3:**
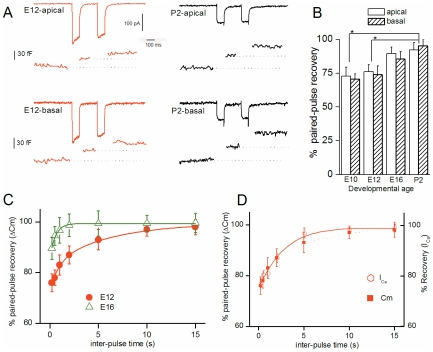
Kinetics of RRP replesnishment increase during development. (**A**) Recording examples of I_Ca_ and ΔC_m_ responses during a paired-pulse protocol (two consecutive 100 ms steps from -90 mV to -10 mV separated by 200 ms) at E12 and P2 in apical and basal tall HCs. (**B**) Comparative paired-pulse recovery (% of first response) at E10 (n = 7), E12 (n = 8), E16 (n = 7) and P2 (n = 4). Recordings were made with an intracellular Ca^2+^ buffer of 0.5 mM EGTA. Note a larger depression in immature pre-hearing HCs E10–E13 as compared to P2 mature HCs. Asterisks indicate a statistical difference with p<0.01. (**C**) Comparative kinetics of RRP recovery in E12 and E16 apical HCs when varying the inter-pulse time in the paired-pulse protocol. In immature E12 HCs, data points were best fitted with two exponentials with time constants of 800 ms and 6 s (n = 5). In E16 HCs (n = 5), data points were best fitted with a single exponential with time constant of 680 ms. (**D**) During the paired-pulse protocol, in E12 apical HCs, RRP and I_Ca_ showed similar depression or slow kinetics of recovery.

Auditory HCs must be able to quickly replenish the RRP to sustain neurotransmitter release [22], [24]. To address this issue, we examined the developmental changes associated with the rate of vesicle supply to the RRP in apical and basal HCs using a 100 ms paired-pulse depolarization protocol (Fig. 3). In both basal and apical immature HCs, exocytosis showed marked paired-pulse depression that decreased with maturation from 25% in E12 to 5% in P2 HCs (Fig. 3A–3B). When varying the inter-pulse interval, approximately 95% of the RRP was restored within ∼ 6 s at E12, and ∼ 0.7 s at E16 (e.g. time constants (s) E12: best fit with dual exponential function with τ_1_ = 0.8±0.1 and τ_2_ = 6.1±1.4 (n = 5); E16: best fit with a single exponential function τ = 0.7 ±0.1 (n = 5), *p<0.05*; Fig. 3C). Notably, marked paired-pulse depression or inactivation was also observed for I_Ca_ in immature E12 HCs as compared to mature P2 HCs. The kinetics of RRP recovery in E12 HCs paralleled the time course of I_Ca_ recovery (Fig. 3D), indicating that ΔC_m_ paired-pulse depression was mainly due to I_Ca_ inactivation in immature HCs. By contrast, mature HCs reconstituted ∼ 95% of the RRP within less than 200 ms and showed almost no I_Ca_ inactivation (e.g. P2: 95±2%, (n = 4), *p<0.001*; table 2). We did not refine the kinetics of paired-pulse recovery below 200 ms because capacitance measurements are altered by Ca^2+^ tail currents below this time frame.

**Table 2:** Vesicle replenishment characteristics in apical HCs.

	E10	E12	E16	P2
**Paired pulse depression 100ms pulse - 200ms interval**				
**0.5 mM EGTA**		**25±9%**		**5±2%**
**2 mM EGTA**	not tested	**30±5%**	not tested	**90±2%**
**RRP replenishment time constant**				
τ**_1_**	**850ms**	**800 ms**	**680 ms**	**<200ms**
τ**_2_**	**7s**	**6s**	**-**	
**Number of ribbons per cell**	**0.3±0.5**	**2.1±1.6**	**3.1±1.4**	**9.3±2.2**

**Figure 4:**
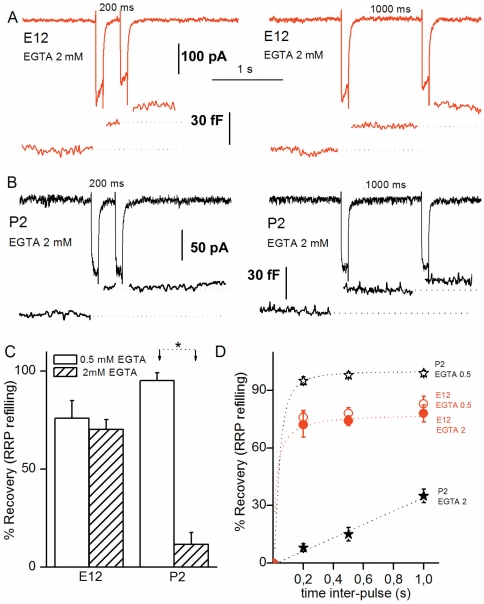
Rate of vesicle replenishment becomes highly sensitive to intracellular EGTA with maturation. A) Two paired-pulse recordings of I_Ca_ and ΔC_m_ in response to two consecutive 100 ms depolarizing steps (as in Fig 3) separated by either 200 ms or 1 s in an E12 apical tall HC using 2 mM intracellular EGTA. (B) Two paired-pulse recordings of I_Ca_ and ΔC_m_ as in A but in mature P2 apical HCs. Note the strong depression in ΔC_m_ after the 2^nd^ pulse. (C) Comparative RRP recovery after 200 ms inter-pulse using 0.5 or 2 mM intracellular EGTA in E12 (n = 5) and P2 (n = 5) apical HCs. Asterisk indicates statistical significance with p<0.01. (D) Comparative kinetics of RRP recovery using 0.5 or 2 mM intracellular EGTA in mature P2 (n = 5) or immature E12 (n = 5) apical HCs.

Next, we compared the rate of vesicle supply to the RRP when using an intracellular recording solution containing 2 mM EGTA instead of 0.5 mM (Fig. 4). Surprisingly, at E12 using either 0.5 mM or 2 mM EGTA, the RRP showed a similar level of paired-pulse depression, indicating that the refilling rate was poorly Ca^2+^-sensitive (Fig. 4A, 4C, 4D). By contrast, reduced intracellular Ca^2+^ availability with high EGTA largely increased paired-pulse depression in mature HCs (e.g. at P2, (mM EGTA, % RRP recovery 200 ms after the first 100 ms-pulse): 0.5, 95±2 (n = 4); 2, 10±2, (n = 4), *p<0.001*), Fig. 4B, 4C, 4D). These results obtained in mature chick HCs are in agreement with previous findings [22] and suggest that the supply of vesicle to the RRP is Ca^2+^-sensitive. The novelty of our findings is that this vesicle supply process in immature HCs and mature HCs shows a different Ca^2+^ sensitivity.

### The efficiency of vesicular recruitment increases during development and allows sustained release

**Figure 5:**
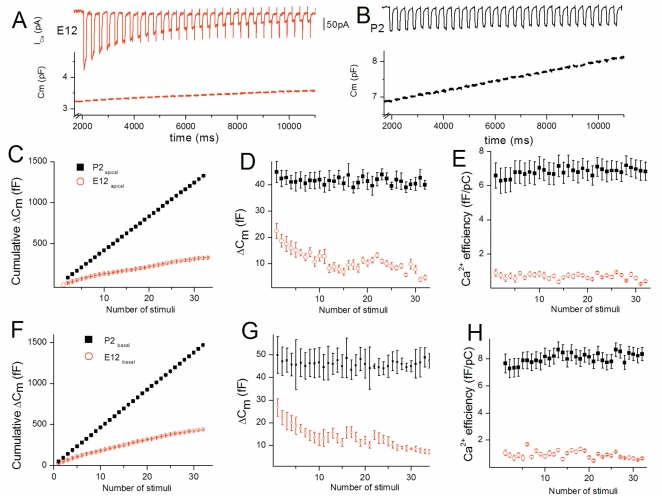
An highly efficient vesicle recruitment allows sustained release in mature HCs. A)Recording examples of C_m_ and I_Ca_ from an E12 apical HC during a train of 100 ms pulses (from -90 mV to -10 mV), each separated by 200 ms. B) Recruitment example evoked in similar conditions in a P2 apical HC. C–D–E) Apical HCs: Comparative mean cumulative ΔC_m_ changes over number of stimuli in E12 (n = 5) and P2 (n = 4) HCs (C). Comparative mean ΔC_m_ changes over number of stimuli (D). Contrary to mature P2 HCs, E12 immature HCs cannot sustain constant exocytosis during the train of stimuli. The comparative Ca^2+^ efficiency (fF/pC) calculated after each stimulus is shown in E. (F, G, H) Summary data comparing cumulative ΔC_m_ changes and Ca^2+^ efficiency at E12 (n = 5), and P2 (n = 4) in basal HCs.

The RRP replenishment was also studied by stimulating the HCs with a train of consecutive brief stimuli consisting of 100 ms depolarizing steps from -90 mV to -10 mV separated by 200 ms (Fig. 5). Cumulative ΔC_m_ responses showed marked depression in immature developing HCs, as indicated by a progressive decrease in ΔC_m_ responses during the repetitive stimuli: In basal high frequency HCs, ΔC_m_ decreased from a mean of 27±4 fF after the first stimulus to 10±3 fF after the 20^th^ one at E12 (n = 5; *p<0.001*; Fig. 5G). Similar depression was observed in E12 low frequency HCs (Fig. 5D). In these immature HCs, the Ca^2+^ efficiency of vesicle release (ΔC_m_/Q_Ca_) from RRP remained constant during the repetitive stimulations (Fig. 5E, 5H), indicating that the depression of exocytosis mainly arose from the marked inactivation of the Ca^2+^ current (Fig. 5A). By contrast, mature P2 HCs from base or apex showed no depression of the RRP, as indicated by a near linear increase in cumulative ΔC_m_ (Fig. 5B–5F). Mature P2 HCs showed constant high Ca^2+^ efficiency in exocytosis (Fig. 5E, 5H) and absence of I_Ca_ inactivation (Fig. 5B).

### Otoferlin expression and number of synaptic ribbons per HC increase with maturation

**Figure 6:**
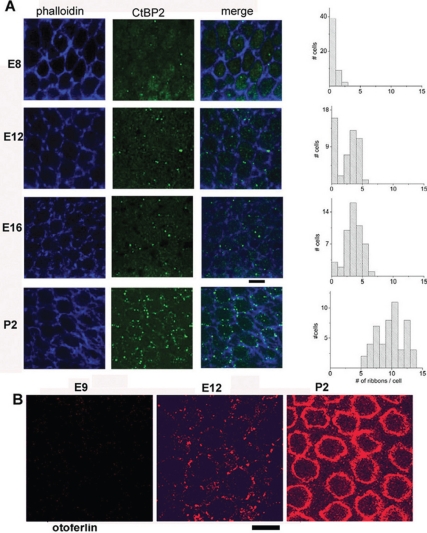
CtBP2 protein (RIBEYE) and otoferlin expression in developing chick HC. A) Surface preparations of the chick basilar papilla at various developmental stages were labeled with CtBP2 (green) and phalloidin antibodies (blue). Confocal images are averaged over 5–9 Z-stack images of 0.4 µm each and taken from the nucleus area to the bottom of the cell. At right, the graphs indicate the corresponding distribution of the averaged number of ctBP2 spots (ribbons) per HCs from 5 basilar papilla in the low frequency region. Lower bar scale indicates10 µm. B) Confocal images showing otoferlin (red) expression at different developmental stages in the apical low frequency region of chick cochlea (E9, E12 and P2). Note the increasing expression of otoferlin at the HC plasma membrane with development.

Several factors could explain the increase in kinetics and Ca^2+^ efficiency of exocytosis in HCs during development: a reduction in the distance between the Ca^2+^ channels and the sites of release at the active zone; a change in the affinity of the Ca^2+^ sensor that controls membrane fusion; or a reduction in the diffusion barrier of Ca^2+^ ions at the site of release. We found that the average number of ribbons per HC largely increased with development from 0.3±0.5 (n = 50) at E8, 2.1±1.6 (n = 50) at E12, 3.1±1.4 (n = 50) at E16 and 9.3±2.2 (n = 50) at P2 (Fig. 6A and table 2). These results indicate a positive correlation between Ca^2+^ efficiency in exocytosis and the number of synaptic ribbons in HCs. Our observations are in agreement with the recent finding that the synaptic ribbons contribute largely to synaptic neurotransmission by facilitating high rates of exocytosis, while their absence significantly compromise the temporal resolving power of the auditory system [25], [26].

We then used the recently characterized monoclonal antibody HCS-1 [17] to explore the expression of the calcium sensor otoferlin during development of chick basilar papillae. Otoferlin was weakly expressed in HCs at embryonic stages earlier than E10 and then increased with development to reach a maximum level at E18-P2 (Fig 6B). At these late stages of development, HCS-1 immunolabeling was largely distributed at the plasma membrane from the apical part of the HCs (below the cuticular plate) to the lower end of the HCs (synaptic area). It is to be mentioned that, in absence of a true control as in mouse knock out for the otoferlin gene [5], [7], we cannot ascertain that the HCS-1 labeling is entirely specific. However, it is to be noted that both the plasma membrane labeling and the developmental increase of HCS-1 labeling matched very well the recent results obtained in mouse HCs [7].

## Discussion

This report characterizes the functional changes occurring during progressive maturation of the HC synaptic machinery in a precocial post-hearing vertebrate, the chick, where sound-evoked cochlear nuclei activity can be measured as early as E11 *in ovo* [12], [13]. Concomitantly to an increased expression of ribbons and otoferlin, exocytosis of chick HCs progressively displayed faster kinetics and higher Ca^2+^ efficiency with maturation. Similar changes have been shown in HCs of pre-hearing animals such as mouse and gerbil [9], [23]. Our study demonstrates for the first time that vesicle supply and RRP release undergo a parallel maturation to allow mature HCs to sustain high rates of exocytosis. In addition, we show that vesicle recruitment is highly Ca^2+^-dependent in mature chick HCs, in agreement with previous findings [22]. Notably, a constant vesicle trafficking from a reserve pool has also been recently proposed to be Ca^2+^-dependent in turtle auditory HCs [27].

Remarkably, immature chick HCs displayed significant depression in exocytosis during repetitive brief stimuli or paired-pulse stimulation, while mature HCs showed little RRP depression. This exocytotic depression in immature HCs is likely due in part to the rapid inactivating property of the Ca^2+^ current at this developmental stage. Indeed, the Ca^2+^ current and the RRP showed similar kinetics of recovery during paired-pulse stimulations. Notably, while the Ca^2+^ current of mature chick HCs is mainly driven by non-inactivating dihydropyridine-sensitive L-type Ca^2+^ channels [28], [29], immature chick HCs (in addition to L-type channels) transiently express fast inactivating T-type Ca^2+^ channels, [16] and unpublished data. Furthermore, L-type Ca^2+^ currents of immature HCs display strong calmodulin-mediated calcium-dependent inactivation [30]. Therefore, Ca^2+^ current inactivation leading to RRP depression could partially explain the transient rhythmic temporal discharge pattern of the auditory nerve fibers observed in young kittens [11] and in chicken embryos [14], [31]. These immature animals show high threshold low frequency hearing and are unable to maintain a sustained steady-state response to long duration tone bursts [32].

In agreement with [22], we found that RRP replenishment, but not initial release, was diminished by using 2 mM EGTA instead of 0.5 mM in mature chick HCs. These results suggest that the release sites are less than 200 nm from Ca^2+^ entry, while the reloading sites extend farther than 200 nm. Similar Ca^2+^ regulation of vesicle replenishment has been shown at the cone ribbon synapses of the retina [33]. In HCs of the amphibian papilla, recovery from paired-pulse depression has recently shown to be ultrafast and also dependent on Ca^2+^ [34]. The most intriguing result of our study was the observation that the RRP recovery of immature HCs, unlike mature chick HCs, was not sensitive to 2 mM intracellular EGTA. This may reflect differences in the distance of the stock of vesicular supply from the release sites and Ca^2+^ entry during development. A larger and wider extrasynaptic distribution of Ca^2+^ channels at the early stage of development, as shown in immature mouse HCs [35], could place Ca^2+^ entry closer to the refilling machinery (reserve pool of vesicles) in immature HCs and in turn make vesicle replenishment less sensitive to EGTA. Notably, we found a positive correlation between the increasing number of ribbons with maturation and the efficiency of vesicle supply and release. Our results obtained in developing HCs are in good agreement with those obtained in a recent study using transgenic mice lacking the presynaptic scaffold protein bassoon, an essential element to dock the ribbon to the active zone [26]. The latter study concluded that the ribbon is essential for organizing Ca^2+^ channels and vesicles in the synaptic active zone in order to promote efficient vesicle replenishment.

In addition to an increased number of ribbons during maturation, the organization of a different Ca^2+^-dependent vesicle supply may also progressively take place in mature HCs. Otoferlin, which is considered to be a high affinity Ca^2+^ sensor that directly triggers SNARE-membrane fusion i*n vitro* [4] and regulates Ca^2+^-evoked membrane fusion at the ribbon synapse of both cochlear [5] and vestibular HCs [36], could also regulate a Ca^2+^-dependent vesicle supply at a large distance from Ca^2+^ entry. Indeed, we found that the expression of otoferlin increases at the right period of cochlear maturation when vesicle replenishment becomes efficient, suggesting a progressive engagement of this Ca^2+^ sensor in the ribbon active zone. By progressively replacing other Ca^2+^ sensors such as synaptotagmins during development [7], otoferlin may facilitate vesicle supply and release at the mature HC ribbon synapse.

Contrary to mature auditory HCs, the ribbon synapses of the retina do not express otoferlin [17], [37] and display pronounced paired-pulse depression that is attributable to a limiting slow replenishment of vesicles [38], [39]. Indeed, RRP recovery in retinal bipolar neurons displays rather slow kinetics (τ ∼ 4 to 8 s) spanning the range of what we found in immature HCs. Our present study, showing a concomitant developmental onset of a fast Ca^2+^-sensitive vesicle supply and otoferlin expression, suggests that this multi-C2 protein may also act as a Ca^2+^ sensor for the recruitment of vesicles located far from the release sites and Ca^2+^ channels, and probably farther than 200 nm as suggested by its sensitivity to 2 mM EGTA. This hypothesis is also reinforced by the phenotype of the *pachanga* mouse model, which carries a missense mutation in the C2F domain of otoferlin, and where the replenishment process of synaptic vesicles is affected independently of RRP fusion [6].
